# Genetic Factors of Renin–Angiotensin System Associated with Major Bleeding for Patients Treated with Direct Oral Anticoagulants

**DOI:** 10.3390/pharmaceutics14020231

**Published:** 2022-01-19

**Authors:** Jeong Yee, Tae-Jin Song, Ha-Young Yoon, Junbeom Park, Hye-Sun Gwak

**Affiliations:** 1 College of Pharmacy and Graduate School of Pharmaceutical Sciences, Ewha Womans University, Seoul 03760, Korea; yee.j@ewha.ac.kr (J.Y.); dymphna@ewha.ac.kr (H.-Y.Y.); 2 Department of Neurology, Ewha Womans University Seoul Hospital, Ewha Womans University College of Medicine, Seoul 07804, Korea; knstar@ewha.ac.kr; 3 Division of Cardiology, Department of Internal Medicine, Ewha Womans University Mokdong Hospital, Ewha Womans University College of Medicine, Seoul 07985, Korea

**Keywords:** direct oral anticoagulants, bleeding, renin–angiotensin system, polymorphisms, pharmacogenomics

## Abstract

The purpose of this study was to identify the renin–angiotensin system (RAS)-related genetic factors associated with bleeding and develop the bleeding risk scoring system in patients receiving direct oral anticoagulants (DOACs). This study was a retrospective analysis of prospectively collected samples from June 2018 to May 2020. To investigate the associations between RAS-related genetic factors and major bleeding, we selected 16 single nucleotide polymorphisms (SNPs) from five genes (namely, *AGT*, *REN*, *ACE*, *AGTR1*, and *AGTR2*). Multivariable logistic regression analysis was employed to investigate the independent risk factors for bleeding and to develop a risk scoring system. A total of 172 patients were included in the analysis, including 33 major bleeding cases. Both old age (≥65 years) and moderate to severe renal impairment (CrCl < 50 mL/min) increased the risk of bleeding in the multivariable analysis. Among RAS-related polymorphisms, patients carrying TT genotype of rs5050 and A allele of rs4353 experienced a 3.6-fold (95% CI: 1.4–9.3) and 3.1-fold (95% CI: 1.1–9.3) increase in bleeding, respectively. The bleeding risk increased exponentially with a higher score; the risks were 0%, 2.8%, 16.9%, 32.7%, and 75% in patients with 0, 1, 2, 3, and 4 points, respectively. Although this study is limited to a retrospective study design, this is the first study to suggest RAS-related genetic markers and risk scoring systems, including both clinical and genetic factors, for major bleeding in patients receiving DOAC treatment.

## 1. Introduction

Direct oral anticoagulants (DOACs) are widely used for anticoagulation therapy to reduce the risk of stroke or systemic embolism in patients with non-valvular atrial fibrillation and to prevent or treat several types of thromboembolic events, including venous thromboembolism [1,2]. Two classes of DOACs are currently available—direct thrombin inhibitors (dabigatran) and direct factor Xa inhibitors (rivaroxaban, apixaban, and edoxaban) [3].

Bleeding is the most severe and common complication of anticoagulation treatment [4]. In a large retrospective observational study, the incidence rate of major bleeding was 3.5%, 5.3%, and 3.5% per person-year in the dabigatran, rivaroxaban, and apixaban groups, respectively [5]. Although all DOACs showed lower risks of intracranial hemorrhage than warfarin, they had similar or higher risks of gastrointestinal (GI) bleeding [6,7,8,9]. Several meta-analyses using real-world studies have also shown similar results, supporting the previous randomized controlled trial (RCT) findings [10,11].

Unlike warfarin, DOACs are administered at fixed doses with no need for routine monitoring [12]. There is considerable interindividual variability in pharmacokinetic and pharmacodynamic responses to DOACs, which can be explained by several clinical factors (e.g., age, weight, and renal dysfunction) [13,14]. Nevertheless, it still remains largely unexplained; genetic factors may play an important role. As most pharmacogenomic studies of DOACs have been restricted to the genes involved in the metabolism and transport of DOACs, such as *CES1* and *ABCB1* [15,16], the goal of this study was to discover novel genetic associations with DOAC responses.

The renin–angiotensin system (RAS), which consists of renin (REN), angiotensinogen (AGT), angiotensin I-converting enzyme (ACE), and angiotensin receptors (AGTR1 and AGTR2), is largely involved in coagulation and fibrinolysis pathways [17,18]. For example, angiotensin II induces the expression of plasminogen activator inhibitor type 1, whereas ACE degrades bradykinin, which stimulates the production of tissue-type plasminogen activator [19]. In addition, several studies have shown that RAS polymorphisms are related to several cardiovascular diseases such as coronary heart disease, stroke, and hemorrhage [20,21]. As with previous research, we hypothesized that RAS could affect bleeding complications. Therefore, this study aimed to identify the RAS-related genetic factors associated with major bleeding and to develop the bleeding risk scoring system in Korean patients receiving DOACs.

## 2. Materials and Methods

### 2.1. Study Patients

The present study was a retrospective analysis of prospectively collected samples from June 2018 to May 2020. It was conducted at Ewha Womans University Mokdong Hospital, Severance Hospital of Yonsei University College of Medicine, and Seoul National University Hospital. As the incidence of major bleeding was low, a case-cohort study approach was applied; patients at Ewha Womans University Mokdong Hospital were enrolled as the cohort, and additional cases were recruited from all hospitals. This study was approved by the Institutional Review Board (IRB) of each hospital in accordance with the Declaration of Helsinki (IRB numbers: 2018-04-006, 4-2018-0823, and 1811-076-9850, respectively). Written informed consent was obtained from all patients before they were enrolled.

The eligible patients for this study were those who had received DOACs (apixaban, edoxaban, rivaroxaban, or dabigatran) for at least 3 months. Patients were excluded if they met the following criteria: (1) <18 years old, (2) had thromboembolic or infarction-related events during the follow-up period, (3) experienced bleeding that was minor or unverified by health professionals while on treatment, or (4) refused consent. Patients who experienced major bleeding during the DOAC treatment were defined as cases, while controls were those without any documented bleeding events during the DOAC therapy. Major bleeding was defined according to the criteria of the International Society on Thrombosis and Haemostasis (ISTH) [22].

Data collection was performed by reviewing the electronic medical records, including sex, age, body weight, creatinine clearance (CrCl), DOAC type, alcohol use, smoking, comorbidity, concomitant medication, and any history of myocardial infarction, stroke, transient ischemic attack, thromboembolism, or bleeding. The modified HAS-BLED (excluding liable international normalized ratio (INR)) scores were calculated using the established definitions of the different risk factors [23].

### 2.2. Genotyping Methods

To investigate the associations between RAS-related genetic associations and major bleeding, we selected five candidate genes (*AGT*, *REN*, *ACE*, *AGTR1*, and *AGTR2*). Based on minor allele frequencies and linkage disequilibrium (LD) in Asian populations [24,25] and previous findings [26], a total of 16 single nucleotide polymorphisms (SNPs) were chosen: rs7079, rs699, rs5050, and rs1112257 of *AGT*; rs2368564 and rs12750834 of *REN*; rs1800764, rs4341, and rs4353 of *ACE*; rs275651, rs2640543, rs5186, and rs5182 of *AGTR1*; and rs1403543 of *AGTR2*.

Genomic DNA was extracted from patients’ blood samples using the QIAamp DNA Blood Mini Kit (QIAGEN, Hilden, Germany) or saliva samples using OraGene-600 kits (DNA Genotek, OTT, Canada) and PrepIT reagents (DNA Genotek, OTT, Canada). Genotyping was performed using the TaqMan SNP genotyping assay (Applied Biosystems, Foster City, CA, USA) or SNaPshot Multiplex Kits (Applied Biosystems, Foster City, CA, USA).

### 2.3. Statistical Analysis

The unpaired *t* test was used to compare the continuous variables between patients who experienced bleeding and those who did not. For categorical variables, the chi-squared test and Fisher’s exact test were used. Both dominant and recessive models were applied for analysis, and we selected the most appropriate model in consideration of both effect size and statistical significance. LD coefficients (r^2^) for each SNP pair were calculated by haploview 4.2 [25], and the haplotype was constructed for the SNP pairs with an r^2^ ≥ 0.8 using PLINK. To identify the independent risk factors for bleeding, multivariable logistic regression analysis with backward elimination was performed using variables with *p* < 0.05 in the univariate analysis, along with age and sex. Odds ratios (ORs) and adjusted ORs with 95% confidence intervals (CI) were calculated from univariate and multivariable analyses, respectively. The model fit of the prediction model was assessed by the Hosmer–Lemeshow goodness-of-fit test. For the development of a risk scoring system, each coefficient from the logistic regression model was divided by the smallest one and rounded to the nearest integer. The discrimination of the risk score was further evaluated by calculating the area under the receiver operating characteristic curve (AUROC). All the statistical analyses were performed using SPSS v20.0 (IBM Corp., Armonk, NY, USA), and a *p* value of <0.05 was considered statistically significant.

## 3. Results

Among the 224 patients enrolled, 52 were excluded for the following reasons: 4 patients had been treated with DOACs for <3 months, 23 patients had experienced thromboembolic or infarction-related events, and 25 patients had nonmajor bleeding. Accordingly, 172 patients were included, among whom there were only 33 cases.

The demographic and clinical characteristics of the cases and controls in the study are shown in Table 1. The median age of the patients was 69 years, ranging from 40 to 94 years. Males comprised 62.8% of the patients. Around three-quarters of the patients had hypertension, and one-quarter had diabetes mellitus. Among DOACs, factor Xa inhibitors comprised 90% of the patients. In terms of co-medications, the most common drug class was beta-blockers (77.9%), followed by statins (47.6%) and angiotensin-converting enzyme inhibitors or angiotensin II receptor blockers (40.1%). Old age (≥65 years) and moderate-to-severe renal impairment (CrCl < 50 mL/min) were significant clinical factors for major bleeding. In addition, cases had higher modified HAS-BLED scores than controls (2.3 vs. 1.9, *p* = 0.035).

Table 2 shows the RAS-related genetic associations with major bleeding. Allele frequencies of the SNPs were in consensus with previous observations of Asians (Table S1). Two SNPs, namely, *AGT* rs5050 and *ACE* rs4353, were significantly associated with major bleeding. Patients with TT genotype had more bleeding than those with the G allele for *AGT* rs5050 (25.5% vs. 10.1%, *p* = 0.013). In terms of *ACE* rs4353, GG genotype carriers showed lower risks of bleeding compared to A allele carriers (10.4% vs. 23.5%, *p* = 0.038). Similarly, in the haplotype analysis, patients carrying the homozygotes of the C-G haplotype of rs4341 and rs4353 had lower major bleeding than the others (10.0% vs. 23.3%, *p* = 0.045).

Multivariable logistic regression analysis showed that age, CrCl, *AGT* rs5050, and *ACE* rs4353 were independent risk factors for bleeding (Table 3). After adjusting for covariates, patients aged 65 years and older had a 3.1-fold (95% CI: 1.1–8.5) increased risk of bleeding compared to those younger than 65 years, whereas patients with a CrCl < 50 mL/min showed a 4.2-fold (95% CI: 1.4–12.3) higher risk of bleeding than those with a CrCl ≥ 50 mL/min. For rs5050 and rs4353, patients carrying the TT genotype of rs5050 and the A allele of rs4353 experienced a 3.6-fold (95% CI: 1.4–9.4) and a 3.1-fold (95% CI: 1.1–9.3) increase in bleeding, respectively. The Hosmer–Lemeshow test showed that the fitness of the final model was satisfactory (χ^2^ = 2.388, 7 degrees of freedom, and *p* = 0.935).

**Figure 1:**
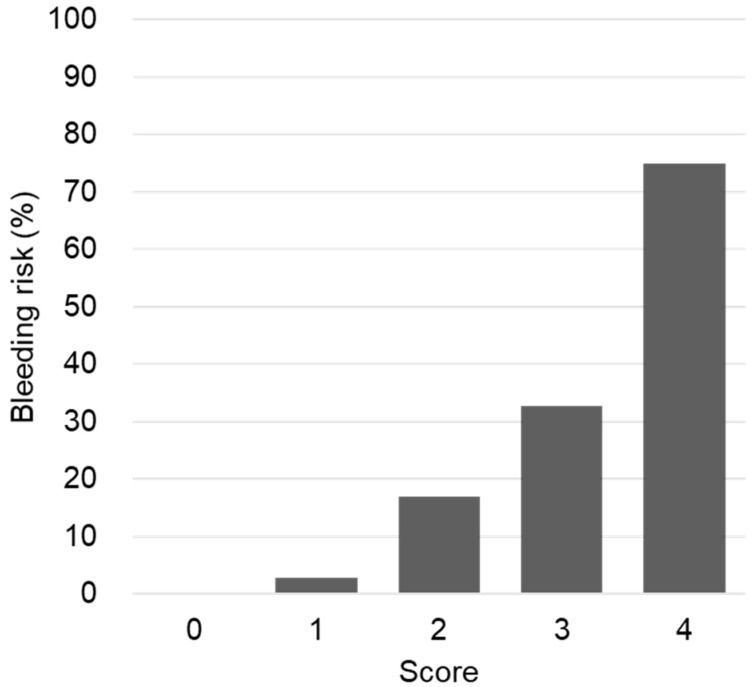
The bleeding risk according to the risk scores.

Based on the final model, we developed the risk scoring system to predict bleeding. We assigned one point to each of four variables in the model: age ≥ 65 years, CrCl < 50 mL/min, AGT rs5050 TT, and ACE rs4353 AA, AG. The total score ranged from 0 to 4. Figure 1 shows the bleeding risk at each point. The bleeding risk increased exponentially with higher scores; the risks were 0%, 2.8%, 16.9%, 32.7%, and 75.0% in patients with 0, 1, 2, 3, and 4 points, respectively. The AUROC was 0.738 (95% CI: 0.649–0.826, *p* < 0.001).

## 4. Discussion

This study demonstrated that old age and moderate-to-severe renal impairment increased the bleeding risks. Among RAS-related genetic polymorphisms, *AGT* rs5050 and *ACE* rs4353 were significantly associated with bleeding. The risk score, which incorporated both clinical and genetic factors, predicted bleeding well, with an AUROC of 0.738.

AGT, a precursor of angiotensin, is a rate-limiting of angiotensin I production and is therefore a key component of the RAS [17,27]. As RAS has been involved in cardiovascular physiology and pathology, *AGT* polymorphisms often confer susceptibility to cardiovascular diseases such as hypertension and myocardial infarction [21,28]. Among the several SNPs, rs5050, which is located in the promoter region of *AGT*, is one of the most extensively studied polymorphisms [29]. Several studies have demonstrated that variant allele of rs5050 increases the risks of hypertension [30], coronary artery aneurism [31], and left ventricular hypertrophy [32]. In addition, the variant allele was related to higher risks of aspirin-related bleeding ulcers and warfarin-induced minor bleeding, compared to the wild-type allele [33,34]. However, in our study, DOAC-treated patients carrying the wild-type allele of rs5050 were prone to major bleeding. This may be due to the organ-specific effects of variants. In general, this variant is known to increase the transcription rate by 20–40% [35]. However, according to the GTEx portal [36], the wild-type allele of rs5050 increased *AGT* expression, especially in the brain. Perdomo-Pantoja et al. also suggested that patients with wild-type allele homozygotes had a poor prognosis of astrocytoma with increased AGT levels [37]. Considering previous findings, the higher risk of bleeding in wild-type allele carriers of rs5050 in this study was attributable to the high proportion (around 60%) of cerebrovascular hemorrhage in the case group. When we limited the cases to cerebrovascular hemorrhage, the OR doubled to 6.5. However, further studies are needed, as there have been limited studies involving the brain.

ACE, a main enzyme of the RAS, converts biologically inactive angiotensin I to active angiotensin II, which binds to and activates AGTR1 and AGTR2 [17]. The ACE gene is highly polymorphic and contributes to interindividual differences in ACE activity [38]. Although rs4353, one of the significant SNPs associated with bleeding in this study, is an intronic variant, it may be in the binding site for transcription factors and thereby has functional effects [39]. According to the GTEx portal, the variant allele of rs4353 is related to a decreased expression of *ACE* [36]. Zhang et al. also showed that the serum ACE level was lower in patients with variant homozygotes of rs4353 than in patients with the wild-type allele [40]. The wild-type allele of rs4353 is known as a risk factor for hypertension [30]. In addition, the wild-type allele of rs4353 is known to increase the warfarin-related minor bleeding [34]. Taken together, the wild-type allele of rs4353 may lead to an increased expression of *ACE* and susceptibility to bleeding.

Advanced age is one of the known risk factors for major bleeding, and several anticoagulant-associated bleeding risk scoring systems (e.g., HAS-BLED, HEMORR2HAGES, and ATRIA) include age as a prognostic factor [23,41]. According to a large cohort study (*n* = 39,539), patients aged 65–74 and those aged ≥ 75 experienced a 1.9-fold (95% CI: 1.4–2.5) and a 3.5-fold (95% CI: 2.7–4.6) increased risk of bleeding, respectively, compared to those aged 18–64 [42]. Our study also showed that old age was significantly associated with an increased bleeding risk.

Another risk factor associated with major bleeding is moderate to severe renal impairment (CrCl < 50 mL/min). Chronic renal impairment causes a variety of pathophysiological changes, including both hyper- and hypocoagulability [43]. Although several anticoagulant-associated bleeding risk scoring systems have considered renal impairment, most of them include severe conditions. For example, HAS-BLED, HEMORR2HAGES, and ATRIA only included chronic renal failure and/or dialysis among the risk factors [23,41]. However, according to Apostolakis et al., patients with CrCl < 60 mL/min had a 1.6-fold higher risk of major bleeding than those with CrCl ≥ 60 mL/min [44]. Yao et al. have also reported that patients with CrCl < 50 mL/min experienced a 1.3-fold (95% CI: 1.0–1.6) higher risk of bleeding than those with CrCl ≥ 50 mL/min [42].

Despite there being several bleeding risk scoring systems for anticoagulants, most were developed and validated in patients receiving vitamin K antagonists [41]. According to Yao et al., the performance of bleeding risk scoring systems in DOAC-treated patients was modest with AUROCs ranging from 0.60 to 0.68 [42], which needs to be further investigated. In addition, genetic factors have rarely been considered [41]. Among several risk scoring systems, HEMORR2HAGES considered genetic factors (i.e., *CYP2C9* SNPs) as a bleeding risk factor [45]. However, this can be applied to warfarin, but not to DOACs, due to their different metabolic pathways. Our risk scoring system, which incorporates clinical and genetic factors, could be a novel approach to predict DOAC-related major bleeding.

This study may still have some limitations that should be considered when interpreting the results. The first limitation is the retrospective design, and the second limitation is the relatively small sample size. Third, we did not consider other gene polymorphisms, such as SNPs of *ABCB1* and *CYP3A4/5*, which could affect the DOAC pharmacokinetics. Further prospective large cohort studies are needed to validate our findings and to evaluate the effects of other pharmacokinetic gene polymorphisms. Despite this limitation, this is the first study to suggest RAS-related genetic markers and risk scoring systems based on both clinical and genetic factors for major bleeding in patients receiving DOAC treatment.

## Figures and Tables

**Table 1:** Baseline characteristics of study patients.

Characteristics	Cases (*n* = 33)	Controls (*n* = 139)	*p*
Sex			0.073
Male	20 (60.6)	88 (63.3)	
Female	13 (39.4)	51 (36.7)	
Age (y)			0.021
<65	6 (18.2)	55 (39.6)	
≥65	27 (81.8)	84 (60.4)	
Body weight (kg)			0.416
<60	11 (33.3)	36 (26.3)	
≥60	22 (66.7)	101 (73.7)	
Creatinine clearance (mL/min)			0.013
<50	8 (25.0)	11 (8.1)	
≥50	24 (75.0)	124 (91.9)	
Systolic blood pressure (mmHg)	127.6 ± 17.9	131.4 ± 20.0	0.334
Diastolic blood pressure (mmHg)	73.0 ± 11.2	77.3 ± 14.4	0.118
Modified HAS-BLED			
<2	6 (18.2)	52 (37.4)	0.036
≥2	27 (81.8)	87 (62.6)	
Type of DOAC			1.000
Direct thrombin inhibitors	4 (12.1)	16 (11.5)	
Factor Xa inhibitors	29 (87.9)	123 (88.5)	
Dose of DOAC ^a^			0.572
Underdose	13 (39.4)	44 (31.7)	
Standard dose	20 (60.6)	93 (66.9)	
Overdose	0 (0.0)	2 (1.4)	
Alcohol	7 (21.2)	45 (32.8)	0.193
Smoking	5 (15.6)	24 (17.5)	0.798
Comorbidity			
Chronic heart failure	7 (21.2)	34 (24.5)	0.694
Hypertension	24 (72.7)	96 (69.1)	0.680
Diabetes mellitus	9 (27.3)	33 (23.7)	0.671
Dyslipidemia	6 (18.2)	26 (18.7)	0.474
Hepatic abnormality	1 (3.0)	2 (1.4)	0.474
Renal abnormality	2 (6.1)	1 (0.7)	0.095
Previous stroke/TIA/thromboembolism	17 (51.5)	62 (44.6)	0.474
Previous bleeding events	4 (12.1)	13 (9.4)	0.745
Comedication			
Antiplatelet drugs	3 (9.1)	7 (5.0)	0.407
ACEI/ARB	16 (48.5)	53 (38.1)	0.275
Beta-blocker	23 (69.7)	111 (79.9)	0.206
Calcium channel blocker	8 (30.8)	30 (22.1)	0.337
Diuretics	14 (42.4)	36 (25.9)	0.060
Statins	20 (60.6)	62 (44.6)	0.098

^a^ Standard dose was defined according to the FDA-approved labeling. ACEI: angiotensin converting enzyme inhibitor; ARBs: angiotensin II receptor blocker; DOAC: direct oral anticoagulant; TIA: transient ischemic attack.

**Table 2:** Factors associated with bleeding in patients receiving direct oral anticoagulants.

Gene Polymorphism	Minor Allele Frequency	Grouped Genotypes	Cases (*n* = 33)	Controls (*n* = 139)	*p*
*AGT*	0.12	GG	27 (81.8)	111 (81.0)	0.916
rs7079		GT, TT	6 (18.2)	26 (19.0)	
*AGT*	0.18	AA, AG	9 (27.3)	42 (30.4)	0.721
rs699		GG	24 (72.7)	96 (69.6)	
*AGT*	0.36	TT, TC	25 (75.8)	123 (89.1)	0.083
rs11122576		CC	8 (24.2)	15 (10.9)	
*AGT*	0.20	TT	26 (78.8)	76 (55.1)	0.013
rs5050		TG, GG	7 (21.2)	62 (44.9)	
*REN*	0.19	CC	21 (63.6)	92 (67.2)	0.701
rs2368564		CT, TT	12 (36.4)	45 (32.8)	
*REN*	0.37	GG	12 (36.4)	54 (39.1)	0.769
rs12750834		GA, AA	21 (63.6)	84 (60.9)	
*ACE*	0.45	CC, CT	27 (81.8)	90 (65.2)	0.065
rs1800764		TT	6 (18.2)	48 (34.8)	
*ACE*	0.42	GG, GC	26 (78.8)	89 (64.5)	0.116
rs4341		CC	7 (21.2)	49 (35.5)	
*ACE*	0.45	AA, AG	28 (84.8)	91 (66.4)	0.038
rs4353		GG	5 (15.2)	46 (33.6)	
*AGTR1*	0.13	TT	26 (78.8)	105 (77.2)	0.845
rs275651		AT, AA	7 (21.2)	31 (22.8)	
*AGTR1*	0.15	AA, AG	8 (24.2)	36 (26.1)	0.828
rs2640543		GG	25 (75.8)	102 (73.9)	
*AGTR1*	0.25	CC, CT	16 (48.5)	55 (39.9)	0.366
rs5182		TT	17 (51.5)	83 (60.1)	
*AGTR1*	0.04	AA	32 (97.0)	125 (91.2)	0.467
rs5186		AC, CC	1 (3.0)	12 (8.8)	
*AGTR2*	0.34	GG, GA	17 (51.5)	49 (35.8)	0.096
rs1403543		AA	16 (48.5)	88 (64.2)	

**Table 3:** Multivariable analysis to identify predictors for bleeding in patients receiving direct oral anticoagulants.

Variables	Unadjusted OR (95% CI)	Adjusted OR (95% CI)
Female	1.12 (0.51–2.44)	
Age (≥65 years)	2.95 (1.14–7.60)	3.08 (1.12–8.46) *
CrCl (<50 mL/min)	3.76 (1.37–10.32)	4.16 (1.41–12.31) *
*AGT* rs5050 TT	3.03 (1.23–7.45)	3.56 (1.35–9.43) *
*ACE* rs4353 AA, AG	2.83 (1.03–7.81)	3.14 (1.06–9.31) *

CI: confidence interval; OR: odds ratio. * *p* < 0.05.

## Data Availability

The data presented in this study are available on request from the corresponding author. The data are not publicly available due to privacy or ethical restrictions.

## Supplementary Materials

The following supporting information can be downloaded at: www.mdpi.com/article/10.3390/pharmaceutics14020231/s1, Table S1: Allele frequencies of single nucleotide polymorphisms (SNPs) analyzed in the study.
