# Supplementary material for: Genetic Factors of Renin–Angiotensin System Associated with Major Bleeding for Patients Treated with Direct Oral Anticoagulants
Source: Pharmaceutics. 2022 Jan 19;14(2):231. doi: 10.3390/pharmaceutics14020231 (PMC8877686; doi:10.3390/pharmaceutics14020231)
Supplement: Supplementary file 1 [file pharmaceutics-14-00231-s001.zip › pharmaceutics-1558876-supplementary.pdf]

pharmaceutics

MDPI

Supplementary Materials: Genetic Factors of Renin–Angiotensin System Associated with Major Bleeding for Patients Treated with Direct Oral Anticoagulants

Jeong Yee, Tae-Jin Song, Ha Young Yoon, Junbeom Park and Hye Sun Gwak

Table S1. Allele frequencies of single nucleotide polymorphisms (SNPs) analyzed in the study.

Gene	SNP	Allele change	Variant allele frequency of study patients (Koreans)	Variant allele frequency of Asians[a]
AGT	rs7079	G > T	0.10	0.12
AGT	rs699	A > G	0.83	0.84
AGT	rs11122576	T > C	0.37	0.35
AGT	rs5050	T > G	0.22	0.17
REN	rs2368564	C > T	0.19	0.22
REN	rs12750834	G > A	0.37	0.36
ACE	rs1800764	C > T	0.55	0.65
ACE	rs4341	G > C	0.57	0.69
ACE	rs4353	A > G	0.54	0.64
AGTR1	rs275651	T > A	0.12	0.14
AGTR1	rs2640543	A > G	0.86	0.80
AGTR1	rs5182	C > T	0.78	0.67
AGTR1	rs5186	A > C	0.04	0.07
AGTR2	rs1403543	G > A	0.70	0.63

[a] Haploreg v4.1.
